# Prevalence of Sleep-Disordered Breathing in Prader–Willi Syndrome

**DOI:** 10.1155/2023/9992668

**Published:** 2023-10-26

**Authors:** Ahmed Abushahin, Amal Al-Naimi, Mutasim Abu-Hasan, Rania Arar, M. Lina Hayati, Antonisamy Belavendra, Ibrahim A. Janahi

**Affiliations:** Department of Pediatric Medicine, Sidra Medicine, Doha 26999, Qatar

## Abstract

**Introduction:**

Sleep-disordered breathing (SDB) is common in patients with Prader–Willi Syndrome (PWS). However, the prevalence of SDB varies widely between studies. Early identification of SDB and factors contributing to its incidence is essential, particularly when considering growth hormone (GH) therapy.

**Objectives:**

The aims of the study were to describe the prevalence and phenotypes of sleep-disordered breathing (SDB) in patients with Prader–Willi syndrome (PWS) and to determine the effects of age, gender, symptoms, GH therapy and body mass index on SDB severity.

**Methods:**

This study was a retrospective chart review of all patients with genetically confirmed Prader–Willi syndrome who underwent diagnostic overnight polysomnography (PSG) in the sleep laboratory at Sidra Medicine. Clinical and PSG data of enrolled patients were collected.

**Results:**

We identified 20 patients (nine males, eleven females) with PWS who had overnight sleep polysomnography (PSG) at a median age (IQR) of 5.83 (2.7–12) years. The median apnea-hypopnea index (AHI) was 8.55 (IQR 5.8–16.9) events/hour. The median REM-AHI was 27.8 (IQR 15–50.6) events/hour. The median obstructive apnea-hypopnea index (OAHI) was 7.29 (IQR 1.8–13.5) events/hour. The median central apnea-hypopnea index (CAHI) was 1.77 (IQR 0.6–4.1) events/hour. Nineteen patients (95%) demonstrated SDB by polysomnography (PSG) based on AHI ≥1.5 events/hour. Nine patients (45%) were diagnosed with obstructive sleep apnea (OSA). Three patients (15%) were diagnosed with central sleep apnea (CSA). Seven patients (35%) were diagnosed with mixed sleep apnea. No correlations were observed between AHI and age, gender, BMI, symptoms, or GH therapy. However, REM-AHI was significantly correlated with BMI (*P*=0.031).

**Conclusion:**

This study shows a high prevalence of SDB among our patients with PWS. Obstructive sleep apnea was the predominant phenotype. BMI was the only predictor for high REM-AHI. Further studies of large cohorts are warranted to define SDB in PWS and design the appropriate treatment.

## 1. Introduction

Prader–Willi syndrome (PWS) is a rare genetic disorder characterized by the absence of the expression of the paternally inherited genes in chromosome 15 q11–13 region [1]. The estimated prevalence of PWS is one in 10,000–25,000 live births [2]. Patients with PWS can have multisystem abnormalities that include neurodevelopmental delay, growth retardation, endocrine and metabolic disturbances, and behavioral disorders that vary with age. During infancy, the main clinical features of PWS include hypotonia, feeding difficulties, and poor growth. Patients develop hyperphagia from childhood and onward due to hypothalamic dysfunction and consequent morbid obesity. Other manifestations include hypogonadism, psychomotor delay, hypothyroidism, and short stature [1, 2].

Sleep-related breathing disorders (SDBs) are common and potentially serious complications of PWS that can affect patients at any age. Multiple studies have reported a high prevalence of SDB among individuals with PWS ranging between 44 and 100%, compared to a prevalence of 2-3% in the general population [1–3]. Craniofacial dysmorphology affecting upper airway size, adenotonsillar hypertrophy, obesity, hypotonia, chest wall deformities, and defective ventilatory control due to hypothalamic dysfunction contribute to the overall high prevalence of SDB in PWS [1, 2].

The spectrum of sleep-related disorders seen in PWS includes central sleep apnea (CSA), alveolar hypoventilation, and obstructive sleep apnea (OSA), with OSA being the most dominant form [1, 4]. Other patterns of SDB include altered sleep architecture, excessive daytime sleepiness, and abnormal ventilatory response to hypercapnia and hypoxia [4]. The trajectory of SDB in PWS evolves with age from predominantly CSA in infants to OSA in older children [5]. Since sleep-related disorders in PWS is a major contributor to the increase in morbidity and mortality in children with PWS, surveillance polysomnography (PSG) has been advocated in children with PWS to detect early SDB and promptly provide the necessary treatment [1, 6].

To date, the prevalence, severity, and treatments of SDB among patients with PWS in Qatar have not been reported. Our study aims to describe the prevalence, severity, and treatments prescribed for SDB in patients with PWS who underwent PSG study in the only dedicated pediatric sleep lab in the country. We also aim to evaluate cohort specific clinical characteristics that can potentially contribute to SDB in our population.

## 2. Methods

This is a retrospective chart review of all patients with genetically confirmed PWS who underwent full-night polysomnography (PSG) between September 1^st^, 2019, and July 30^th^, 2022, at Sidra Medicine, which is a tertiary care hospital in the state of Qatar and houses the only pediatric sleep lab in the country.

The electronic medical records were utilized to obtain patients' demographic and anthropometric characteristics including age, sex, height, and weight at the time of PSG. Body mass index (BMI) was calculated as weight (kg)/height (m^2^). BMI *z*-score was calculated using the most recent age and sex specific growth charts published by the World Health Organization (WHO) [7]. Obesity was defined as BMI>90th percentiles or as BMI *z*-score>2. Reported symptoms, history of adenotonsillectomy, and GH treatment were also collected.

PSG studies were conducted in our sleep laboratory using Alice 6LDx system (Philips Respironics, USA) by trained sleep technicians. The following standard PSG measurements were obtained during sleep study: electroencephalogram, electrooculogram, submental and limb electromyogram, electrocardiography, oronasal airflow and temperature, thoracic and abdominal wall movements, percutaneous oxygen saturation (SpO_2_), and body positions. The following parameters were collected from the generated sleep reports: apnea-hypopnea index (AHI), rapid eye movements-related apnea-hypopnea index (REM-AHI), obstructive apnea-hypopnea index (OAHI), central apnea-hypopnea index (CAHI), average oxygen saturation (SpO_2_%), lowest SpO_2_%, percentage of total sleep time (TST) spent with SpO_2_ <90%, and average end-tidal carbon dioxide (EtCO_2_). All events and parameters were scored according to the American Academy of Sleep Medicine (AASM) Staging and Scoring Manual V2.5 2018 [8].

The diagnosis of sleep-disordered breathing (SDB) was confirmed if the patient had AHI ≥1.5. Central sleep apnea (CSA) was considered if a patient had CAHI of ≥1.5 events/hour and OAHI of <1.5 events/hour. Obstructive sleep apnea (OSA) was considered if the patient had OAHI ≥1.5/hour and CAHI <1.5 events/hour. Mixed sleep apnea was considered if patient had both CAHI ≥1.5 and OAHI ≥1.5. For patients with OSA, the severity was categorized based on AHI as follows: [1] mild OSA if AHI was 1.5–4.9, [2] moderate OSA if AHI 5–9.9, and [3] severe OSA if AHI ≥10. Alveolar hypoventilation was considered if ETCO_2_ >50 mmHg for more than 25% of TST. A decrease in SpO_2_≥3% from baseline defined O_2_ desaturation events. Nocturnal hypoxemia was defined as sleep time≥2% of TST with a SpO_2_<90%.

### 2.1. Statistical Analysis

Demographic, anthropologic, and clinical characteristics were summarized as the mean and standard deviation (SD) for symmetrically distributed continuous variables and median and range for the skewed continuous variables. Scatterplots were drawn between age and BMI with AHI, and Spearman's rank correlation was used to determine the correlation between the skewed variables. Positively skewed variables (AHI and REM-AHI) were log-transformed. The relationships between logged outcome variables (AHI and REM-AHI) and age, gender, BMI, symptomatic, and GH therapy were assessed using linear regression analysis. Predictor variables' selection in regression analysis was carried out based on the clinical importance. All statistical analyses were performed using STATA IC/16.0 (StataCorp LLC, College Station, Texas, USA).

## 3. Results

A total of 23 patients with genetically confirmed PWS who had diagnostic PSG from September 1^st^, 2019, until July 30^th^, 2022, were identified and included. Twenty patients had complete PSG data (nine males and eleven females). The median (IQR) age at the time of PSG was 5.83 years (2.7–12). Median (IQR) BMI (kg/m^2^) was 25.89 (20.1–36.3), and median (IQR) BMI *z*-score was 3.77 (2.7–4.6) (Table 1). According to patients' medical records, eight patients (40%) reported a history of snoring, eight patients (40%) reported combined symptoms of snoring and witnessed sleep apnea, and four patients (20%) were asymptomatic.

The median sleep efficiency for all patients was 83.1% (IQR 76.8–88.1). The median sleep latency was 12.35 (IQR 2.9–48.6) minutes. Wakefulness after sleep onset (WASO) was 51.2 (IQR 31–80.5) minutes. The median REM% was 20.85% (IQR 17.8–32.7). The median REM latency was 64.5 (IQR 55.5–127) minutes. Eight patients had sleep latency less than eight minutes. Two patients had REM latency less than 15 minutes. The median AHI was 8.55 (IQR 5.8–16.9) events/hour. The median REM-AHI was 27.8 (IQR 15–50.6) events/hour. The median OAHI was 7.29 (IQR 1.8–13.5) events/hour. The median CAHI was 1.77 (IQR 0.6–4.1) events/hour. The mean oxygen saturation (SpO_2_) was 95.45% ± 2.9, and the average minimum SpO_2_ was 78.6% ± 7.1%. The median percentage of time spent with SpO_2_ < 90% was 0.58 (IQR 0.1–2.9) (Table 2). Six patients had nocturnal hypoxemia. The mean of the peak ETCO_2_ was 52 ± 8.2 mmHg; three patients were diagnosed with hypoventilation.

Nineteen out of the 20 patients (95%) had SDB. Nine patients (45%) had obstructive sleep apnea (OSA), three patients (15%) had central sleep apnea (CSA), and seven patients (35%) had mixed apnea. Sleep-disordered breathing was considered mild in three patients (15%), moderate in eight patients (40%), and severe in eight patients (40%) (Table 2).

Multiple regression analysis did not show any significant correlation between AHI, and all examined clinical variables (i.e., age, gender, BMI, symptoms, and GH therapy). On the other hand, REM-AHI was significantly correlated with BMI (*P*=0.031) (Table 3).

Only seven patients (35%) were previously treated with growth hormone. Six patients with OSA were treated with positive airway pressure support (CPAP or BiPAP). One patient had moderate OSA, and the other five had severe OSA. One patient with moderate OSA was treated with night-time O_2_ by nasal cannula. However, six patients with moderate OSA and three with severe OSA received no treatment by the time of the study. All the three patients who were diagnosed with hypoventilation were treated with BiPAP.

## 4. Discussion

In our patient population of PWS, we found very high prevalence of sleep-disordered breathing reaching 95%. The predominant form of SDB observed was OSA. To lesser extent, central sleep apnea and mixed apneas were noted. The true prevalence of SDB in children with PWS has been challenging to determine because of methodological variations, small sample sizes, and age differences among studies. Despite this wide variation, the reported prevalence of SDB in PWS is high, ranging between 44% and 100% [1, 2]. In one meta-analysis of 14 studies, Sedky et al. estimated average prevalence of OSA in children with PWS to be 79.91% [9].

The most commonly reported phenotypes of SDB in PWS are OSA, CSA, and hypoventilation [5]. However, the type of SDB varies with age. CSA is more frequently found among infants than older children with PWS [1, 4]. In one study, the reported prevalence of CSA among infants<1 year of age was high (53%) compared to OSA (11%) [10]. In another study, prevalence of CSA was reported to be 71.8% among children<2 years compared to 25% in children>2 years of age [3]. The underlying mechanism of this high CSA in infants with PWS is not entirely understood. It has been proposed that hypotonia, immaturity of the brain stem, and hypothalamic dysfunction in infants could be the underlying etiology [2]. On the other hand, OSA is much more common in older children with PWS. Cohen et al. reported significant predominance of OSA (52%) compared to CSA (5%) among children with PWS who are>2 years old [11]. In our patient cohort, the dominant SDB phenotype was OSA (45%), while CSA was observed in only 15%. The higher prevalence of OSA in our population is probably related to the high-median age of the included patients (5.8 years).

The discrepancy seen in the prevalence of reported phenotypes of SDB among different cohorts could be related to differences in criteria used to define the phenotypes of SDB, in particular central sleep apnea (CSA) and mixed sleep apnea. Previously published PSG studies in PWS use CAHI≥5 events/hour as the criteria for diagnosis of CSA arguing that CAHI of up to 5 event per hour is expected in normal children more than one year of age [10, 12]. Using this definition of CAHI>5 events/hour, only one patient (5%) had CSA and three patients (15%) had mixed apnea in our cohort. However, some of our patients had abnormal SDB, defined as AHI≥1.5 events/hour, with CAHI≥1.5 but <5 events/hour. We argue that these patients cannot be considered completely normal as suggested by previous studies but probably have mild abnormal control of breathing. Future longitudinal studies are needed to determine if these patients develop disease progression and have higher long-term morbidity.

Among our patients with OSA, three patients (15%) had mild disease, eight patients (40%) had moderate disease, and eight patients (40%) had severe disease. Others reported that among individuals with OSA, 53.07% had mild OSA, 22.35% moderate OSA, and 24.58% severe OSA [9]. Unlike CSA, OSA is a well-known and potentially serious sleep-disordered breathing in PWS. Canora et al. reported OSA in 92.9% of patients with PWS. The mean obstructive apnea-hypopnea index (OAHI) in their cohort was 7.6 ± 4.2 events/h [12]. Our patient cohort had median OAHI of 7.29 events/hour.

In our patients, the mean SpO_2_ was 95.45% ± 2.9, with a minimum SpO_2_ of 78.6% ± 7.1%. Previous studies reported similar mean SpO_2_ of 96.6 ± 0.6% and nadir of 77.2 ± 10.2% [13]. Pavone et al. reported mean peak PtcCO_2_ of 47.9 ± 8.1 mmHg [14]. In our patients, the mean peak ETCO_2_ was higher at 52 ± 8.2 mmHg. However, only three of our patients were diagnosed with alveolar hypoventilation defined as ETCO_2_ ≥50 mmHg for more than 25% of TST.

There are limited studies investigating sleep architecture in individuals with PWS. Our patients demonstrated a reasonably normal sleep architecture with a mean sleep efficiency of 83.1%. The median REM% was normal at 20.85% (14.9–41.5) minutes, and the median REM latency was 64.5 (4.5–254.5) minutes. Similarly, Lin et al. reported the REM latency of 67.4 ± 30.0 minutes and REM percentage of 21.1 ± 5.7% [13]. Nevertheless, larger sample size studies of similar age cohorts are required to precisely define the characteristics of the sleep architecture among patients with PWS.

Obesity in general has been recognized as a predictor of OSA by several studies [15, 16]. OSA risk increases by 12% for every 1 kg/m^2^ increase in BMI above the 50th percentile among healthy children [15]. In addition to the impact of obesity on respiratory mechanics, it also worsens the abnormal ventilatory responses to hypoxia and hypercapnia in PWS patients [16]. As expected, patients in our cohort were overweight. The BMI *z*-score was 3.77 (IQR 2.7–4.6), consistent with the reported high prevalence of obesity in patients with PWS [3]. However, the link between BMI and SDB in PWS is controversial. Some studies showed increased SDB with increasing BMI, while others failed to demonstrate this correlation [1, 12, 17]. Sedky et al.'s meta-analysis revealed that OSA in PWS patients increased with greater BMI (*r* = 0.34, *P*=0.018) [9]. In contrast, our study did not show a correlation between BMI and AHI, but there was significant correlation between BMI and REM-AHI (*P*=0.031). Further studies are required to determine if sleep-related breathing abnormalities in PWS are related to obesity alone or to other factors such as hypotonia, control of breathing, or facial dysmorphism.

Several treatment modalities for SDB in PWS have been proposed based on SDB pattern and severity. Infants with CSA are generally treated with nocturnal supplemental oxygen [2, 11]. In children with moderate to severe OSA, adenotonsillectomy is recommended. Several studies have demonstrated partial response or no response to adenotonsillectomy in PWS [1, 9, 18]. Strategies for weight loss can lead to improvement of OSA in obese PWS patients [1, 4]. In patients with persistent moderate to severe OSA, continuous positive airway pressure ventilation is a suitable alternative [4]. Treatment with GH improves height growth, body composition, lean muscle mass, and subsequently improving muscle strength. Therefore, most studies favor GH treatment for SDB in PWS [1, 4, 19]. However, emerging evidence raises concern of worsening OSA after GH therapy. Treatment with GH is associated with high insulin-like growth factor 1 (IGF-1) levels which may lead to enhanced growth of upper airway lymphoid tissues, including adenotonsillar hypertrophy which may contribute to OSA within first two years of therapy [1, 4]. Because of the reported potential worsening of OSA after GH therapy [20, 21], consensus guidelines recommend a screening PSG before initiation and within 3–6 months after the start of GH therapy in all children with PWS [22]. Our study included only few patients who were already on GH therapy. No correlation between GH treatment and SDB has been demonstrated. However, the limited sample size may influence this observation.

Sleep studies were performed in our patients based on PWS diagnosis regardless of symptoms. History of snoring and/or sleep apnea did not correlate with AHI or REM-AHI which supports the recommendation to screen patients with PWS for SDB even in case of absence of symptoms.

## 5. Conclusion

Our study shows a high prevalence of SDB in PWS independent of symptoms supporting the recommendation for screening all individuals with PWS patients using polysomnography. Obstructive sleep apnea was the predominant phenotype of SDB. Central apnea is less frequent which could be due to patients' age. However, CSA in PWS is probably underestimated in previously published studies due to use of high threshold in the diagnostic criteria for CSA, which needs further investigation.

## Figures and Tables

**Table 1:** Clinical characteristics of the patients with PWS (*n* = 20).

Characteristics	All patients
Age, years^∗^	5.83 (**2.7–12**)
Male/female, *n*	9/11
BMI (kg/m^2^)^∗^	25.89 (**20.1–36.3**)
BMI *z*-score^∗^	3.77 (**2.7–4.6**)
History of snoring, *n* (%)	8 (40%)
History of snoring and apnea, *n* (%)	8 (40%)
History of adenoidectomy, *n* (%)	4 (20.0)
History of tonsillectomy, *n* (%)	2 (10.0)
History of GH therapy, *n* (%)	7 (35.0)

BMI, body mass index; GH, hormone therapy. Numerical variables are expressed as ^∗^median (IQR) and categorical variables as frequencies and percentages. The table provides descriptive data for the entire population. The bold values represent the median and interquartile range.

**Table 2:** PSG data of the patients with PWS (*n* = 20).

PSG variables	All patients
Sleep efficiency (%)^b^	83.1 (**76.8–88.1**)
Sleep latency (minutes)^b^	12.35 (**2.9–48.6**)
REM latency (minutes)^b^	64.5 (**55.5–127**)
WASO (minutes)^b^	51.2 (**31–80.5**)
REM (%) of TST^b^	20.85 (**17.8–32.7**)
AHI (events/hour)^b^	**8.55 (5.8–16.9)**
OAHI (events/hour)^b^	7.29 (**1.8–13.5**)
CAHI (events/hour)^b^	1.77 (**0.6–4.1**)
REM-AHI (events/hour)^b^	**27.8 (15–50.6)**
Sleep apnea types, *n* (%)	
CSA	3 (15.0)
OSA	9 (45.0)
Mixed	7 (35.0)
Categories of OSA, *n* (%)	
Mild	3 (15.0)
Moderate	8 (40.0)
Severe	8 (40.0)
Mean sleep SpO_2_, (%)^a^	95.45 ± 2.9
SpO_2_ nadir (%)^a^	78.6 ± 7.1
% of TST with SPO_2_ <90% (%)^b^	0.58 (**0.1–2.9**)
Peak ETCO_2_ (mmHg)^a^	52.0 ± 8.2
Respiratory support, *n* (%)	7 (35.0)

Data are expressed as mean ± standard deviation or median (IQR). Categorical variables are expressed as frequencies and percentages. WASO, wakefulness after sleep onset; AHI, apnea-hypopnea index; OAHI, obstructive apnea-hypopnea index; CAHI, central apnea-hypopnea index; CSA, central sleep apnea; OSA, obstructive sleep apnea; TST, total sleep time; ETCO_2_, end-tidal CO_2_; SpO_2_, O_2_ saturation. The table provides descriptive data for the entire population. The bold values represent the median and interquartile range.

**Table 3:** Regression analysis for AHI and REM-AHI (univariate analysis) with age, BMI, symptoms, GH therapy and AHI, REM-AHI in patients with PWS.

Variables	Logged AHI		Logged REM-AHI	
	*e* ^*β*^ (95% CI)	*p* value	*e* ^*β*^ (95% CI)	*p* value
Age, years	1.01 (0.9, 1.1)	0.680	1.05 (0.9, 1.1)	0.170
Gender (male)	1.41 (0.6, 3.3)	0.415	0.99 (0.4, 2.3)	0.999
BMI (kg/m^2^)	1.02 (0.9, 1.0)	0.239	1.03 (1.1, 1.1)	0.031
GH therapy (yes)	1.01 (0.4, 2.5)	0.989	0.98 (0.4, 2.3)	0.952
History of snoring (yes)	1.70 (0.6, 4.9)	0.302	1.97 (0.7, 5.3)	0.161
History of apnea (yes)	1.83 (0.8, 4.2)	0.148	2.06 (1.0, 4.4)	0.063
Reported adenoid/tonsil hypertrophy (yes)	0.75 (0.3, 2.2)	0.576	1.08 (0.4, 3.1)	0.872

BMI, body mass index; GH, hormone therapy. Estimates of effect size are presented as odds ratios and 95% confidence intervals as exponentiated *β*-coefficients (*e*^*β*^) for the continuous logged variables for AHI and REM-AHI. Exponentiated *β*-values indicate the percentage change in the outcome per unit change in the predictor variable.

## Data Availability

The data that support the findings of this study are available from the corresponding author upon reasonable request.
